# Somatic Mutation Accumulations in Micropropagated Cannabis Are Proportional to the Number of Subcultures

**DOI:** 10.3390/plants13141910

**Published:** 2024-07-11

**Authors:** Kristian Adamek, Andrew Maxwell Phineas Jones, Davoud Torkamaneh

**Affiliations:** 1 Department of Plant Agriculture, University of Guelph, Guelph, ON N1G 2W1, Canada; kadamek@uoguelph.ca (K.A.); amjones@uoguelph.ca (A.M.P.J.); 2 Département de Phytologie, Université Laval, Québec, QC G1V 0A6, Canada; 3 Institut de Biologie Intégrative et des Systèmes (IBIS), Université Laval, Québec, QC G1V 0A6, Canada; 4 Centre de Recherche et d’Innovation sur les Végétaux (CRIV), Université Laval, Québec, QC G1V 0A6, Canada; 5 Institute Intelligence and Data (IID), Université Laval, Québec, QC G1V 0A6, Canada

**Keywords:** genotyping-by-sequencing, genetic diversity, micropropagation, somatic mutations, Cannabis

## Abstract

Advancements in micropropagation techniques have made it easier to produce large numbers of cannabis clones, but these methods may also introduce genetic instability over successive generations. This instability often manifests as somaclonal variation, characterized by the progressive accumulation of genetic mutations or epigenetic alterations with each subculture. In this study, we examined how mutations accumulate in cannabis clones subjected to 6–11 subcultures. Using genotyping-by-sequencing, we identified 9405 polymorphic variants across 70 clones. The analysis revealed a correlation between the number of subcultures and the frequency of these mutations, revealing that genetic changes accumulate over successive subcultures despite clones sharing the same chronological age. Furthermore, we evaluated the functional impacts of accumulated mutations, with particular attention to implications on gene function and overall plant health. While rare, 14 high-impact variants were identified in genes that are important for plant development. Notably, six variants were also found in genes related to cannabinoid and terpene synthesis pathways, potentially affecting the plant’s biochemical composition. These findings highlight the need for genetic assessments in micropropagation protocols, impacting plant breeding and conservation. Understanding genetic variations in clonally propagated plants optimizes practices for stability. Crucial for cannabis and horticultural plants, it emphasizes techniques to prevent genetic decay and ensure viability.

## 1. Introduction

*Cannabis sativa* L. (cannabis), a predominantly dioecious diploid (2*n* = 2*x* = 20), is among the earliest domesticated plants, widely cultivated globally for a diverse range of purposes, including fiber production, seed cultivation, oil extraction, and harnessing its distinct psychoactive and medicinal properties [1]. In recent years, there has been increased recognition, research, and confirmation of its therapeutic compounds with use in various health conditions [2,3,4]. The therapeutic benefits originate mainly from cannabinoids, which interact with the human endocannabinoid system [5]. Two compounds stand out as the most prevalent, extensively researched, and highly sought-after, which are (−)-*trans-*Δ^9^–tetrahydrocannabinol (THC) and cannabidiol (CBD) [6]. Furthermore, cannabis plants produce other biologically active compounds, including terpenes, terpenoids, and flavonoids, which also contribute to the therapeutic properties and may interact in various ways with cannabinoids to modulate the effects, a phenomenon known as the entourage effect [7,8]. The specific medicinal properties of cannabis are closely tied to its distinct composition, and maintaining consistency in plant production is vital for delivering reliable and predictable outcomes. By ensuring that plants are produced under controlled conditions and with stable genetics, the aim is to minimize variations in chemical profiles and optimize the therapeutic potential and consistency of cannabis.

Given the genetic diversity and the heterozygous nature of cannabis, sexual reproduction leads to significant variation in the progeny, which poses challenges for consistency in the chemical composition of the final product [6]. As a solution, clonal propagation has been widely adopted. This method involves taking cuttings from a selected mother plant, ensuring that the clones replicate the mother plant’s genetic makeup and desired traits, such as cannabinoid profiles. Clonal propagation is not unique to cannabis but is also employed in other plant industries, such as strawberries (*Fragaria × ananassa*), coffee (*Coffea arabica* L.), grape (*Vitis vinifera* L.), potato (*Solanum tuberosum* L.), hop (*Humulus lupulus* L.), and banana (*Musa acuminata*) [9,10,11]. Despite its advantages, clonal propagation undergoes challenges, including space allocation for mother plants and the management of pests and pathogens, which can severely impact plant health and chemical integrity [12,13]. In cannabis, numerous instances of this have been recorded with the hop latent viroid (HLVd) and it has been observed to be widespread in cannabis plants in California, as evidenced by 90% of tissue tests out of 200,000 revealing a positive presence of the viroid [14].

To overcome these challenges, micropropagation via meristem tissue culture has been introduced as a promising alternative. This technique involves cultivating plants in a sterile, controlled environment using specialized culture media, which allows for the efficient production of disease-free and genetically uniform plantlets [15,16]. Through multiple subcultures, explants are multiplied exponentially, which can facilitate mass propagation in relatively short periods of time. For cannabis, one of the primary values of micropropagation is the ability to preserve limited and valuable genetic material in a sterile environment for clean plant programs [17]. This allows for the preservation of elite and rare plant genetics in safe conditions free of diseases and pests. However, micropropagation introduces its own set of challenges, notably the potential for somaclonal variation. Genetic and epigenetic changes can arise during tissue culture, which can alter the plant physiology and morphology, potentially affecting growth and chemical composition [18,19]. Genetic somaclonal variations arise from spontaneous mutations in the genome, while epigenetic somaclonal variations refer to alternations in gene expression and the resulting phenotype without any modifications to the DNA sequence [20,21]. Several factors influence the extent of somaclonal variation, including the micropropagation system, genotype, species, explant source, medium composition, and number of subcultures [21,22,23,24]. Addressing these variations is crucial for maintaining the integrity and uniformity of propagated plants, as evidenced by practices in established horticultural industries, such as those for strawberries (*Fragaria × ananassa*) and bananas (*Musa* spp.), where the number of subcultures is carefully controlled to prevent genetic changes [9,25].

In general, restarting the process with fresh stock material is recommended after a specified number of subcultures. In the case of banana (*Musa* spp.), commercial propagators ideally limit the number of subcultures to five before resetting their stock [25]. While off-types, especially dwarf growth habits, accumulate over subcultures, they have identified five rounds as an acceptable threshold to balance economic concerns with genetic fidelity. However, in vitro mutation rates vary based on species, growth conditions, and culture media. Specifically, it is thought that one of the main driving forces of somaclonal variation is the common use of plant growth regulators in the medium [26].

Somatic mutations, genetic alterations occurring outside the germline, are not exclusive to plant tissue culture but occur spontaneously throughout an organism’s life, shaping genetic diversity within populations [27]. Adamek et al. [28] found significant genetic diversity within an individual cannabis mother plant, demonstrating the prevalence of somatic mutations in cannabis plants. This finding aligned with anecdotal reports from cannabis producers indicating that clones deteriorated over time. While somatic mutations can occasionally yield beneficial traits, such as bud sports creating new cultivars [29], they are generally harmful and can compromise plant health. Extensive research is ongoing to discover the mechanisms and mutation rates of somatic mutations throughout various species [30,31,32]. The repair systems can address various types of DNA damage, but their efficiency decreases with age, increasing the likelihood of genetic errors and mutation accumulation [33]. Furthermore, it is important to acknowledge that during each instance of cellular division, there exists a chance for an error to occur and for a mutation to arise [34]. In micropropagation, the continuous initiation and rapid growth of new plants within a tissue culture environment (i.e., culture media) increase the likelihood of mutations occurring [35]. While certain mutations can be beneficial for breeding purposes [30], in the case of cannabis cultivation, uniformity is essential, and variations from mutations are a concern. Genetic mutations can impact genes in several ways, ultimately influencing the function of cellular processes, such as cell growth and division, protein structure and function, gene expression, and gene function [36].

In our previously published study, we explored the effectiveness of genotyping-by-sequencing (GBS) compared with simple sequence repeats (SSRs) in assessing the genetic fidelity of micropropagated cannabis clones [37]. The results demonstrated that GBS provided a far more comprehensive analysis, uncovering extensive genetic diversity that the SSR methods could not detect. Building upon these findings, the current research shifts focus from evaluating genotyping methodologies to examining how genetic variations accumulate in cannabis clones over successive subcultures. By utilizing the superior resolution of GBS, as established in our prior work, this study delves deeper into quantifying the mutation accumulation and its potential impact on clonal fidelity and plant health across multiple generations. Our findings revealed that the accumulation of somatic mutations correlated with the number of subcultures and identified several variants with high-impact effects. These results emphasize the importance of monitoring and managing mutations after several rounds of subculturing and suggest the need for optimizing cannabis micropropagation to ensure long-term uniformity.

## 2. Results

### 2.1. Sequencing and Variant Calling

**Figure 1:**
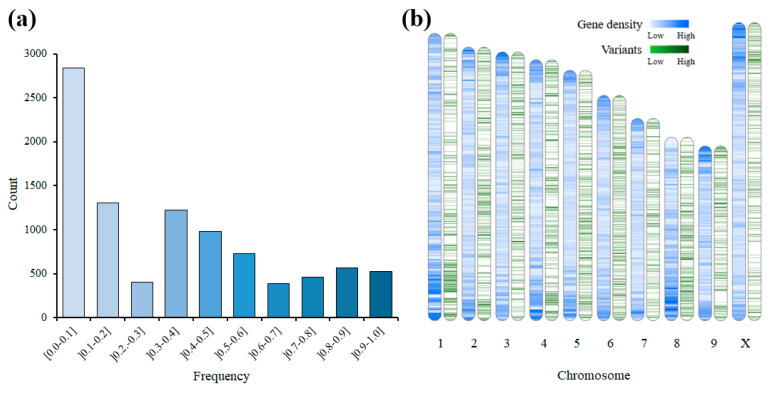
Heterozygous frequencies and chromosomes with gene densities and variants. (**a**) A bar graph showing the frequency of the heterozygous alternative alleles. (**b**) The distribution of gene densities (blue) and variants (green) throughout the cannabis chromosomes.

In the present study, we evaluated the sequencing data and variant calling on cannabis clones that underwent various numbers of subcultures. All the cannabis samples were the same chronological age of 1.5 years old and from a high-THC type-I plant. The GBS procedure yielded a substantial number of raw reads, exceeding 350 million 150-bp paired-end reads in total (Table 1). The sequencing data were then mapped against the cannabis cs10 reference genome, with an average mapping success rate of 96%. After filtering the low-quality variants (as described in the Materials and Methods section), we identified 9405 polymorphic variants, including both single-nucleotide polymorphisms (SNPs) and insertions/deletions (indels), exhibiting a very high depth of coverage (>60X; on average). Moreover, the variants were identified with a transition/transversion ratio of 1.6. The dataset contained a low proportion of missing data, accounting for 39% of the total dataset, which were subsequently imputed. Additionally, we observed an expected prevalence of heterozygous genotypes, with a frequency of 25% across the dataset. Finally, the minor allele frequency analysis revealed an average value of 0.25 across the dataset (Figure 1a).

### 2.2. Distribution and Functional Impact of Variants

To gain insights into the distribution and potential functional implications of the identified variants, we investigated their distribution across the genome. We utilized the cannabis cs10 reference genome to determine the genomic distribution of these variants in relation to the gene density, and an analysis was performed using the cannabis cs10 v2.0 genome (Figure 1b). The variants showed an overall uniform distribution across the genome, with some enrichment observed in genic regions. Specifically, 33.5% of all the variants were located in genic regions, including introns (7.6%), exons (8.5%), and transcriptional variants (17.4%) (Figure S3).

To assess the potential functional impact of the identified variants, we performed an impact categorization and prediction analysis. This allowed us to categorize the variants into high (i.e., variants expected to have a significant effect on protein structure or function), low (i.e., variants deemed mostly harmless or improbable to alter protein function), moderate (i.e., may change the protein effectiveness), and modifier (i.e., non-coding variants that are challenging to predict or lack evidence of impact) impact categories (Table S2). Over 25K effects were predicted with the majority falling into the modifier category (91.48%). The remaining effects were classified as low impact (5.88%), moderate impact (2.59%), and high impact (0.05%). When examining the functional classes, the variants were categorized as missense (32.7%), nonsense (0.3%), and silent (67.0%). Furthermore, we conducted an investigation into the gene description and gene type for the variants predicted to have a high functional impact (Table S3). In addition, a focused search was performed on 44 genes involved in the cannabinoid and terpene pathway (described by [38]), revealing that some of the somatic variants, with a modifier impact, actually occurred in six of these genes (Table 2).

### 2.3. Population Structure

**Figure 2:**
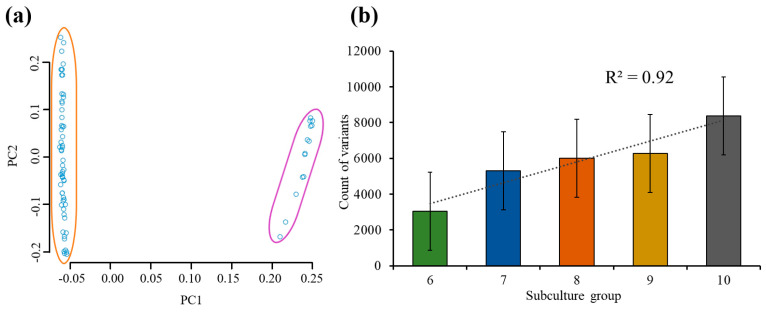
Analysis of the PCA and the number of variants in each subculture group. (**a**) A PCA plot with two primary groupings (orange and purple), which represent the two sister clonal lines. (**b**) A bar graph revealing the impact of consecutive subcultures on the number of variants discovered, with error bars representing the standard deviation.

To better understand the population structure of the variants and any potential patterns, a principal component analysis (PCA) using all the genetic variants was performed. The PCA successfully revealed the presence of the two distinct subpopulations, demonstrating that two sister line groups of micropropagated cannabis had evolved separately with distinct patterns of somatic mutations (Figure 2a). The accurate groupings confirmed the quality of the variants and validated their certainty. The subpopulation on the far-right side of the PCA plot represents the smaller sister line subpopulation, comprising 14 data points (purple outline). In contrast, the primary group of 56 data points is clustered towards the left (orange outline). Furthermore, both subpopulations were spread out primarily vertically in the PCA, indicating the presence of genetic diversity even within the two sister line subpopulations. The non-overlapping nature of the data points in the PCA plot highlights the existence of distinct genetic variations and highlights the potential for genetic diversity within the micropropagated cannabis sister line groups.

### 2.4. Accumulation of Mutations during Subculturing

The accumulation of variants during the process of subculturing was investigated, providing insights into the extent of the genetic changes that occurred with each subculture. As shown in Figure 2b, we observed a significant accumulation of variants as the number of subcultures increased. The initial subculture (i.e., subculture six) served as a reference point, revealing existing genetic diversity with 3K variants identified. Subsequent subcultures led to a steady increase in the number of variants, indicating the accumulation of novel mutations. The accumulation of novel mutations was also revealed from the degree of impact that the variants had, as indicated by the gradual increase in high-impact mutations from seven to 14 (Table S4). The rate of variant accumulation varied in different subcultures, with an average increase of 34% from subculture six to ten. A linear regression coefficient exceeding 0.92 indicated a strong positive association between the number of variants and the number of subcultures. Furthermore, as the number of subcultures increased, the number of sites where the variants were found increased, while the average nucleotide diversity decreased (Table S5). These findings highlight the dynamic nature of the genetic changes occurring during consecutive subculturing and provide valuable insights into the accumulation and impact of novel mutations.

## 3. Discussion

Somatic mutations present a threat to uniformity in the clones of micropropagated cannabis plants and are a key factor contributing to observed somaclonal variations [29]. Consequently, the plant industries utilizing micropropagation for mass production and/or genetic preservation are actively researching and optimizing their systems to mitigate the risks associated with these mutations. Although epigenetics is a major element of somaclonal variants [13,21], our study focuses specifically on somatic mutations and their capacity to exist and carryover in the clones of micropropagated cannabis. Previous studies in cannabis micropropagation have examined genetic diversity in clones and reported minimal or no genetic differences compared with the mother plant [39,40,41]. However, these studies have primarily relied on simple sequence repeat (SSR) markers that only capture a limited portion of the cannabis genome [42]. While SSR markers have been successful in identifying genetic diversity in other plant species [43], it is important to acknowledge that they provide a low-resolution view of the genome. This is significant because research indicates that unique genetic variation is likely to be rare throughout the genome [44]. Therefore, the utilization of advanced DNA sequencing enables the detection of these rare genetic variations and provides a more comprehensive understanding of the genetic landscape.

To our knowledge, no peer-reviewed study utilizing DNA sequencing has been conducted to investigate the relationship of somatic mutations with the number of subcultures in cannabis clones. In this study, we used GBS to examine millions of base pairs, uncovering thousands of variants and demonstrating the presence of numerous somatic mutations within a micropropagated cannabis population. Moreover, the implementation of whole-genome sequencing (WGS) would likely uncover a significantly higher number of somatic mutations. For example, a comparative analysis using the GBS and WGS methods in olive (*Olea europaea* L.) revealed a notable difference in the number of identified SNPs after filtering, with GBS detecting 13 K, while WGS identified 119 K [45]. The inherent limitations of GBS, such as its reduced genomic coverage compared with WGS, may have resulted in an underestimation of the mutation rates and types present in our cannabis clones. While GBS effectively detects a substantial number of variants across the genome, it may miss rare or low-frequency mutations, particularly those occurring outside of the targeted regions. This potential underestimation emphasizes the need for caution when interpreting the mutation rates derived from the GBS data, as the true extent of genomic variability might be greater than reported. Ultimately, it is crucial to view the genetic fidelity of clones as a spectrum of genetic changes instead of a binary description, such as what is frequently done with molecular markers. Furthermore, due to the theory known as Muller’s ratchet [46], the importance is magnified because deleterious somatic mutations accumulate over time/subculture number, thus increasing the probability of negatively impacting valuable traits and compromising the quality of the propagated plants over time. Therefore, a comprehensive understanding of the genome and its mutations is necessary to fully realize the potential threats and establish effective measures for their management.

There are several key mitigation strategies that can minimize the adverse effects of the accumulation of deleterious somatic mutations [35]. First, regular screening of micropropagated plants will facilitate identifying individuals with observable unwanted phenotypic changes and allow the removal of problematic clones [47]. In general, only healthy explants should be maintained, while any unusual growth should be culled during the micropropagation process. This proactive approach enables the maintenance of high-quality plant material. However, it should be noted that important traits, such as cannabinoid profiles in cannabis, may not be readily observable during the clonal stage. Therefore, relying solely on clonal screening does not provide a comprehensive assessment of the desired traits. The implementation of DNA sequencing enables the detection and characterization of somatic mutations, providing insight into their type, frequency, and stability within micropropagated plants very quickly and efficiently. By gaining a deeper understanding of somatic mutations, researchers and breeders can develop appropriate measures to minimize mutation rates, mitigate their impact, and ensure the genetic integrity of cultivated cannabis plants in the future. The rapidly declining costs of DNA sequencing and the increased ease of analyzing large datasets make it a feasible technique that was not available until recently [48,49,50,51]. The location selected for the source material from the mother plant is also crucial, as micropropagation cannabis studies have shown increased success with clones derived from basal regions [23,52]. Similar results have been published with other plant species as well, including English oak (*Quercus robur*) [53], avocado (*Persea americana*) [54], potato (*Solanum tuberosum*) [55], blackberry (*Rubus fruticosus*) [56], and guava (*Psidium guajava*) [57]. These findings suggest that careful selection of the source material contributes to minimizing somaclonal variation. Furthermore, Adamek et al. [28] demonstrated that the genetic mosaicism hypothesis applies to a cannabis mother plant, affirming that spontaneous mutations accumulate and the top contains the most mutations. The optimization of plant growth regulators (PGRs) plays a meaningful role in controlling somaclonal variation. Research has demonstrated that exposing bananas and strawberries to high concentrations of 6-benzylaminopurine (BAP) induced genetic variability and high concentrations of cytokinin in culture media has been considered detrimental to tissue culture media [26,58]. This is particularly interesting in cannabis, where PGRs are not typically employed, as this approach may offer the potential for longer lasting plant material. Lastly, limiting the number of subcultures during the multiplication phase of micropropagation can reduce the opportunities for somatic mutations to arise, preserving genetic fidelity and minimizing somatic mutation accumulation [21]. Among the various mitigation strategies mentioned, limiting the number of subcultures is crucial as it directly reduces the opportunities for mutations by minimizing cell divisions. Similarly, minimizing the use of PGRs is essential, given their potential role as mutagenic agents, which can introduce somaclonal variations [26,58]. Additionally, the implementation of DNA sequencing stands out as a critical tool for monitoring these variations in cannabis micropropagation. Ultimately, employing regular screening, implementing DNA sequencing, selecting an appropriate source material, utilizing proper PGRs, and limiting subcultures are suitable approaches to managing somatic mutations.

Many micropropagation industries have discovered the importance of tracking and controlling the duration and number of subcultures a plant undergoes to minimize undesired variations [9,25]. For example, studies on the in vitro cultivation of *T. hemsleyanum* have revealed that repeated subcultures can lead to increased somatic variations and a decline in the flavonoid content [59]. In a micropropagation study involving cannabis, a morphological observation revealed that the number of nodes and the height significantly decreased after four subcultures [23]. From our results, we documented that more frequent subculturing increases somatic mutations in cannabis using a DNA sequencing approach. These findings enhance the depth of knowledge of previous studies investigating the decline of clones and encourage the development of micropropagation systems to consider. Understanding the potential of accumulating mutations, ensuring the viability and long-term survival of subcultured plants, and optimizing tissue culture techniques are crucial for the advancement of cannabis micropropagation methodologies. It is important to note that accumulating somatic mutations do not diminish the utility of micropropagation but it is rather a factor to consider during the optimization process. Currently, ongoing research is focused on enhancing the cannabis micropropagation process with research into media composition, growth hormones, environmental factors, and lighting conditions [12,16,60,61]. Ultimately, the examination of somatic mutations will likely advance the development of superior procedures, resulting in healthier and more genetically uniform clones. In the future, it is recommended that researchers use various cultivars and utilize DNA sequencing to analyze multiple subcultures, potentially identifying common mutations across the genome. As a result, possibly improving the precision of genotype-to-phenotype correlations for cannabis clones.

Our results show that despite being of the same chronological age, the mutation load was highly correlated with the number/frequency of subcultures. Since somatic mutations typically occur during cell division due to DNA replication errors [62], this is likely a result of the rapid growth after each subculture. As such, for the genetic preservation of elite clonal lines of cannabis, it is recommended to minimize plant growth and subculture frequency. While this was not evaluated in this study, we can presume from these data that slow growth using low-temperature culture conditions or other methods would be ideal to minimize mutation rates. This is common practice in tissue culture-based genetic preservation to reduce mutation rates and labor requirements, as well as to maintain the material in pathogen-free environments [63,64]. In vitro slow-growth conservation has been successfully established for various species, such as fruit trees [65], temperate woody plants [66], horticultural crops [67], and numerous tropical varieties [68]. While it is reasonable to assume that slow growth will reduce mutation rates in cannabis, the ultimate solution for long-term genetic preservation is through cryopreservation, in which the cell cycle is halted, and plant tissues are held in suspended animation indefinitely. This innovative conservation technique has been successfully developed and implemented to preserve a diverse range of species, including strawberry (*Fragaria × ananassa*; [69]), cassava (*Manihot esculenta Crantz*; [70]), potato (*Solanum tuberosum* L.; [71]), and sugarcane (*Saccharum officinarum* L.; [72]). Plants that underwent cryopreservation and regenerated afterwards displayed phenotypically normal characteristics. The data indicate that cryopreserved cells can be preserved for years and even decades. For example, cryobanks facilitate germplasm cryopreservation for several species (e.g., apple, cassava, potato, banana, pear, garlic, and coffee) [73]. While cryopreservation techniques have been reported for cannabis [74], there has been no detailed evaluation of genetic fidelity in this crop. Cryopreservation represents a promising alternative, yet implementing this technique with cannabis presents unique challenges. Moving forward, addressing the genetic fidelity of cryopreserved cannabis tissues will require studying factors such as cryoprotectant selection, cooling rates, and post-thaw recovery protocols to optimize the viability and genetic stability. Additionally, research efforts should focus on validating phenotypic and genotypic consistency post-cryopreservation to ensure the preservation of desirable traits in cannabis cultivars. Cryopreservation is likely the most effective method for long-term genetic preservation. Any practice that slows growth is recommended. In summary, while rapid propagation is essential for meeting market demands, adopting systematic approaches to monitor and manage subculture intervals is pivotal for preserving genetic fidelity in micropropagated cannabis plants. Establishing baseline mutation rates, continuously monitoring genetic stability, and optimizing subculture intervals are key. This comprehensive approach ensures the long-term viability of elite genetic lines in commercial production.

Understanding the potential genes and genomic regions affected by mutations is essential for unraveling the mechanisms underlying somaclonal variation from somatic mutations. In our study, we used GBS to uncover mutations and identify specific genes impacted across the genome of cannabis clones derived using tissue culture techniques. We observed a progressive accumulation of somatic mutations across the genomes, which correlated with the number of subcultures undergone. Although, it is important to recognize the limitations in capturing all the mutations from GBS [75]. Sequencing technology plays a crucial role in capturing a wide range of genomic mutations, and its significance lies in uncovering the influence of previously overlooked regions in the genome, once labelled as “junk DNA” [76]. Genomic regions of non-coding DNA are now recognized as influential regulators of gene expression, which can affect genes through mechanisms such as enhancer elements, long non-coding RNAs, and small RNAs [77]. Therefore, the examination of mutations in these regions is important as they can impact the up- or down-regulation of genes, which can lead to influential changes in the phenotype. Neglecting these regions would overlook an important aspect of genetic variation and its role in controlling traits. Therefore, it is crucial to adopt advanced sequencing techniques capable of capturing a broader spectrum of genomic mutations to achieve a more comprehensive understanding of the genetic fidelity of clones from subculturing.

While this study provides important insights into the accumulation of genetic mutations in micropropagated cannabis clones, it has certain limitations that should be considered. The study was conducted as a survey study, utilizing stock material not initially intended for a research project. As a result, the absence of original mother plants prevented a phenotypic analysis of the clones, which would have provided essential control data to assess the extent of the phenotypic variations resulting from the genetic mutations. Furthermore, the study was not originally designed to correlate specific genetic changes with phenotypic outcomes, limiting the ability to interpret the functional implications of the observed polymorphic variants. To address this limitation, integrating a phenotypic analysis with the genotypic profiling is essential. Future studies should involve assessing traits such as growth characteristics, cannabinoid content, and disease resistance in clones subjected to varying subculture systems. By comparing the phenotypic outcomes with the genotypic data obtained through techniques such as GBS and WGS, researchers can establish correlations between specific mutations and observed variations. While we predicted several high-impact variants, including start-loss, stop-loss, stop-gain, and splice variants, which are likely to significantly affect gene function and thus plant traits, the absence of phenotypic data limits our conclusions. Future studies incorporating a phenotypic analysis would greatly enhance our understanding of how these somatic mutations influence the performance and quality of micropropagated cannabis plants. Such integration is essential for confirming the functional relevance of these mutations and will provide critical insights into optimizing micropropagation protocols to maintain genetic fidelity.

## 4. Materials and Methods

### 4.1. Plant Materials

The cannabis samples utilized in this study were obtained from the in vitro shoots of plants continuously maintained in an established population at the University of Guelph (Guelph, ON, Canada). For a more in-depth description of the methods used, this can be found detailed in Adamek et al. [37]. In brief, the in vitro cannabis plants were initially generated from the seeds of a high-THC type-I cultivar with recognized drug-type properties. These plants were subsequently preserved and propagated following established protocols, as referenced in previous works [39,40,78]. In brief, the plants were maintained on culture media primarily consisting of a DKW Basal Mixture with Vitamins (D2470; Phytotechnology Laboratories, Lenexa, KS, USA), 3% sucrose (*w*/*v*), 0.6% agar (*w*/*v*) (A360-500; Fisher Scientific, Fair Lawn, NJ, USA), 1 mL L^−1^ plant preservative mixture (PPM; Plant Cell Technology, Washington, DC, USA), and adjusted to a 5.7 pH with 1 N NaOH. The majority were also given 5 mg L^−1^ of the insecticide Orthene (AMVAC, Newport Beach, CA, USA) and 1 mL L^−1^ of the antimicrobial PPM as prophylactic treatments. These 78 plants, originating from a newly established in vitro stock plant, were at least 1.5 years old when samples were collected. All the samples used were of the same chronological age, but the number of subcultures they underwent varied from six to 11 from the original explant (Figure S1). Most samples came from a single seedling propagated through nodal explants, with a few from a sister seedling (Figure S2). Fourteen samples were from the sister seedling, and ten were duplicates. After screening, 70 samples were selected for GBS.

### 4.2. Media and Culture Conditions

This population was established through shoot proliferation-based tissue culture techniques using nodal explants, as outlined and explained in the previous work [61]. During the subculturing process, each plantlet was divided into explants, usually consisting of two nodes, which were then placed in GA7 culture vessels (Magenta LLC, Lockport, IL, USA). The explants were typically cultured in basal DKW media following the previously described method [61], although alternative media were employed in a few rare cases. For the specific culture media contents, see Table S1. The culture vessels were maintained in a growth chamber that had controlled temperatures of 25  ±  2 °C, a photoperiod with 16 h of light, and a light intensity of 40  ±  5 μmol m^−2^ s^−1^.

### 4.3. DNA Extraction

To isolate the DNA from the stem tissues, approximately 100 mg of stem tissue was processed with homogenization using a SPEX SamplePrep 1600MiniG^®^ homogenizer (SPEX, Metuchen, NJ, USA) at a speed of 1500 RPM for 2 min. DNA extraction was performed using the NucleoSpin^®^ Plant II Mini Kit (Macherey–Nagel, Düren, Germany) following the manufacturer’s instructions, with a slight modification of including PL1 and RNase A buffers along with the tissue samples during the homogenization step. The quality and concentration of the extracted DNA were assessed using a NanoDrop One spectrophotometer (Thermo Scientific, Waltham, MA, USA). A precise volume of 10 µL, with a concentration of 10 ng µL^−1^, was pipetted into each well of a 96-well semi-skirted polymerase chain reaction (PCR) plate. Afterwards, the plate was sent to the Plateforme d’analyses génomiques [Institut de Biologie Intégrative et des Systèmes (IBIS)], Université Laval (Quebec, QC, Canada), for GBS.

### 4.4. Genotyping-by-Sequencing (GBS)

The 3D-GBS protocol described by de Ronne et al. [79] was employed. The 3D-GBS library was constructed using a combination of three restriction enzymes (*Pst*I/*Nsi*I/*Msp*I) at the Plateforme d’analyses génomiques [Institut de Biologie Intégrative et des Systèmes (IBIS)]. Subsequently, the sequencing was performed on an Illumina NovaSeq 6000 platform at the Centre d’expertise et de services Génome Québec in Montreal, QC, Canada. The sequencing generated a dataset comprising over 377 million paired-end reads, each with a length of 150 base pairs.

### 4.5. Bioinformatic Analysis

The 3D-GBS data utilized in this study can be freely accessed at https://figshare.com/articles/dataset/Genotyping-by-sequencing_GBS_-_Cannbis_clones/24354655 (accessed on 18 October 2023). For the variant calling, the Fast-GBS.v2 pipeline [37,80] was used. In summary, the pipeline included demultiplexing, trimming of the FASTQ files, and mapping against the cannabis cs10 v.2.0 reference genome (GenBank Accession No. GCA_900626175.2), achieving a 96% success rate. Afterwards, the variants, including single-nucleotide polymorphisms (SNPs) and small insertions/deletions (indels), were identified from the mapped reads, and several filtering criteria were applied. Variants were excluded if they were multi-allelic, had an overall read quality (QUAL) score below 20, had a mapping quality (MQ) score below 30, had a read depth below 10, exhibited missing data exceeding 50%, or were monomorphic. To address the missing data, imputation was performed using BEAGLE v5.1 [81], following the methodology outlined by Torkamaneh and Belzile [82]. A raw variant call format (VCF) file containing the genomic variation data was produced. Vcftools v0.1.12b [83] was used to manipulate and analyze the VCF file, which enabled the grouping of samples into subculture categories and the examination of nucleotide diversity. SnpEff v5.0e [84] was employed for the annotation of variants with functional information using the cs10 v.2.0 reference genome database (GenBank Accession No. GCA_900626175.2). Tassel5 v5.0 [85] was used to obtain the genotype information for the alleles, sites, and an overall summary. Lastly, R v4.2.0 R Core Team [86] permitted the performing of a PCA analysis and the analysis of the distribution of variants.

## 5. Conclusions

This study aimed to explore the accumulation of somatic mutations resulting from consecutive subculturing in micropropagated cannabis clones, utilizing the GBS approach. The findings of our investigation demonstrated a consistent increase in somatic mutations with each consecutive round of subculturing, thereby revealing the existence of genetic diversity within the clones and a divergence from the original genotype of the mother plant. The results did not indicate an impending demise of the plants in micropropagation but did offer a potential explanation for the emergence of somaclonal variation in certain plantlets derived using tissue culture techniques. It is crucial to recognize that not all mutations are created equally, and understanding the influence of genetic diversity and somatic mutations on the long-term health and productivity of micropropagated plants is essential for preserving genetic fidelity. Furthermore, it is worth acknowledging that the accumulation of somatic mutations only represents a factor contributing to somaclonal variation, as epigenetic changes and pathogenic factors can also be components. Future investigations should include diverse cultivars of plant species and apply advanced sequencing technologies, such as GBS or WGS, to understand somaclonal variation from somatic mutations comprehensively.

## Figures and Tables

**Table 1:** Summary of the statistical data derived from the genotyping-by-sequencing (GBS) of cannabis clones.

**Total number of raw reads (M)**	377.7
**Average number of reads per sample (M; after trimming)**	4.3
**Average number of mapped reads per sample (M)**	4.3 (96%)
**Total number of polymorphic nucleotide variants**	9405
**Proportion of heterozygous genotypes (%)**	25
**Proportion of missing data (%)**	39
**Average minor allele frequency**	0.25

**Table 2:** Discovered variants in cannabinoid and terpene genes.

Abbreviation	Name	Chromosome	Locus
CMK	CDP-ME kinase	2	LOC115721136
HDS	1-hydroxy-2-methyl-2-(E)-butenyl 4-diphosphate synthase	2	LOC115720893
GPPS.ssu1	Geranyl pyrophosphate synthase small subunit 1	6	LOC115725388
HMGS	Hydroxymethylglutaryl-CoA synthase	5	LOC115716237
PMK	Phosphomevalonate Kinase	5	LOC115716624
MPDC	mevalonate diphosphate decarboxylase	1	LOC115705753

## Data Availability

The dataset supporting the conclusions of this article is available in the FigShare repository: https://figshare.com/articles/dataset/Genotyping-by-sequencing_GBS_-_Cannabis_clones/24354655 (accessed on 18 October 2023).

## Supplementary Materials

The following supporting information can be downloaded at: https://www.mdpi.com/article/10.3390/plants13141910/s1, Figure S1. A dendrogram displaying the records of the primary clonal line samples through their subculture history. Figure S2. A graphic representation of the two sister seeds and subcultures that followed. Table S1. Description of media and hormones/additives used. Figure S3. A pie graph illustrating the distribution of variants percentages across five primary categories, specifically intergenic, up- and downstream regions, genic regions, untranslated regions (UTR), and splice sites. Table S2. Number of variant effects classified by the type and impact. Table S3. Variants in genes that expressed a high-impact effect. Table S4. Distribution of the impact effects across the different number of subcultures. Table S5. Nucleotide diversity and variants values across the different number of subcultures.
