# Supplementary material for: Somatic Mutation Accumulations in Micropropagated Cannabis Are Proportional to the Number of Subcultures
Source: Plants (Basel). 2024 Jul 11;13(14):1910. doi: 10.3390/plants13141910 (PMC11279941; doi:10.3390/plants13141910)
Supplement: Supplementary file 1 [file plants-13-01910-s001.zip › plants-3060351-SI.pdf]

Supplemental Material

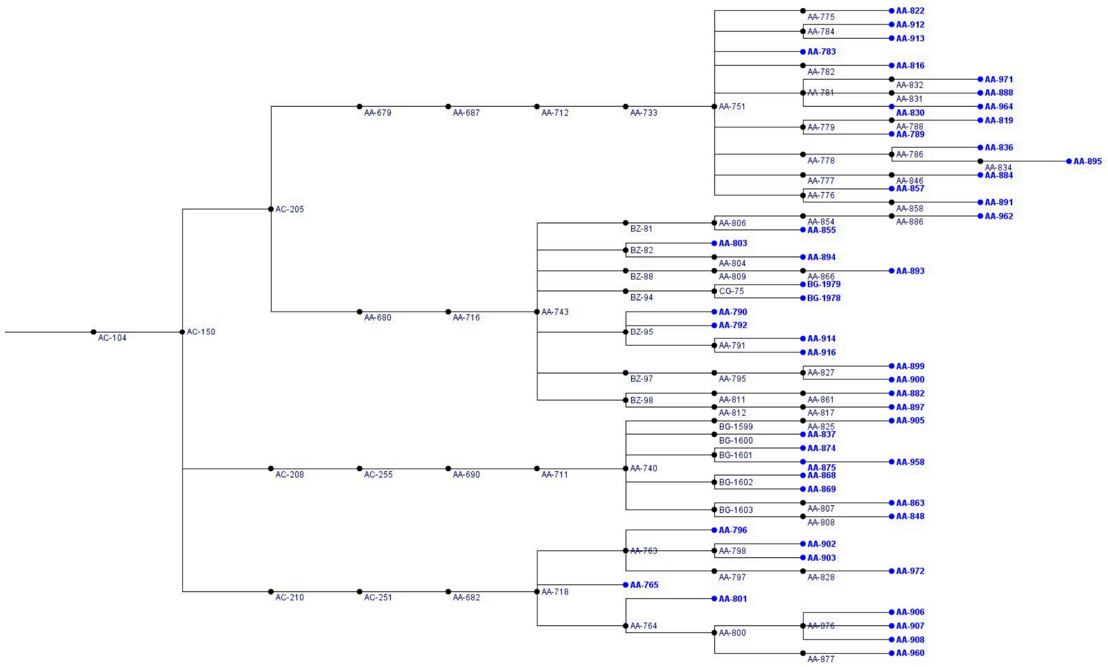

Figure S1. A dendrogram displaying the records of the primary clonal line samples (blue) through their subculture history.

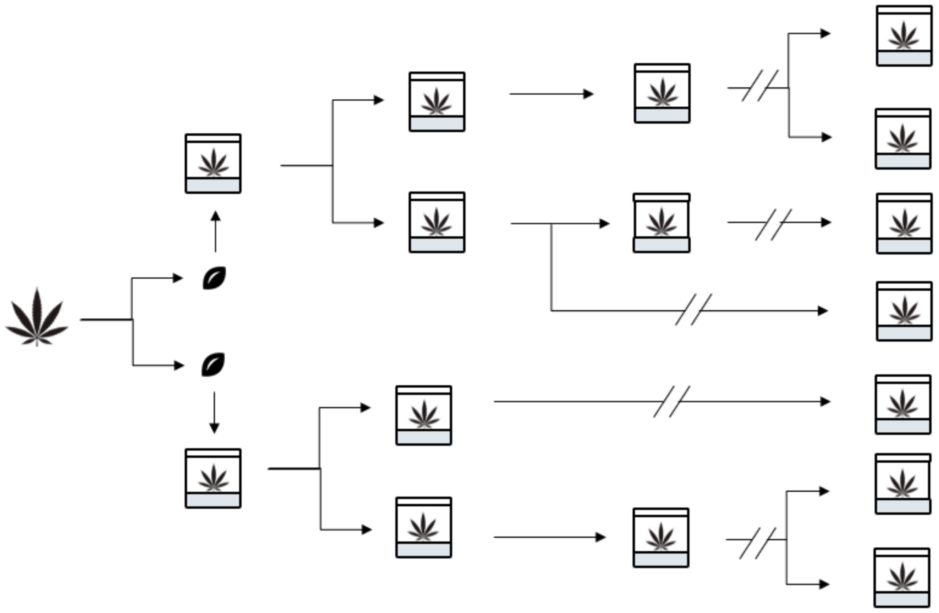

Figure S2. A graphic representation of the two sister seeds and subcultures that followed.

Table S1. Description of media and hormones/additives used.

Sample ID	Medium [a]	Hormones/Additives	Date	Days Elapsed [b]
AC-56	MS	1 mL/L PPM	5/21/2019 14:11	741
AC-104 [c]	MS	20 μM Oryzalin (24hr) + 1 mL/L PPM	7/31/2019 14:07	666
AC-150 [d]	DKW + Vits	1 mL/L PPM	10/2/2019 11:04	605
AC-251	DKW + Vits	0.5 μM TDZ + 1 mL/L PPM	2/12/2020 12:29	472
AA-765	DKW + Vits	5 mg/L Orthene + 1 mL/L PPM	9/28/2020 15:35	243
AA-960	DKW + Vits	3% sucrose + 1 mL/L PPM	5/27/2021 11:45	5

[a] All other samples were on DKW medium
[b] June 1st, 2021, as reference date
[c] Only three early vessels were ever exposed to Oryzalin
[d] Subculture counts began with this vessel

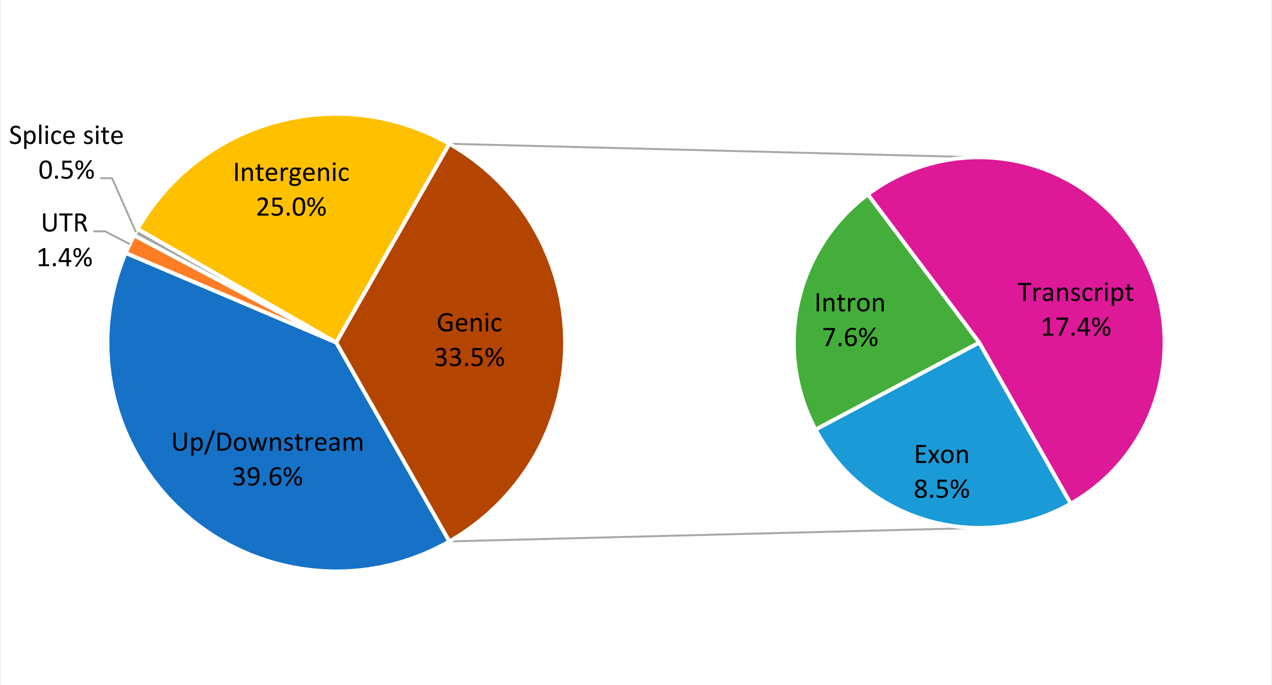

Figure S3. A pie graph illustrating the distribution of variants percentages across five primary categories, specifically intergenic, up- and downstream regions, genic regions, untranslated regions (UTR), and splice sites. The secondary pie graph reveals the composition within the genetic category (intron, exon, transcript).

Table S2. Number of variant effects classified by the type and impact.

Type of effect	Count	Impact
Splice acceptor variant	1	High
Splice donor variant	1	High
Start lost	2	High
Stop lost	4	High
Stop gained	6	High
5 prime UTR premature start codon gain	32	Low
Splice region variant	131	Low
Stop retained variant	2	Low
Synonymous variant	1,374	Low
Missense variant	667	Moderate
3 prime UTR variant	165	Modifier
5 prime UTR variant	154	Modifier
Downstream gene variant	5,325	Modifier
Intergenic region	6,439	Modifier
Intragenic variant	61	Modifier
Intron variant	2,050	Modifier
Noncoding transcript exon variant	168	Modifier
Noncoding transcript variant	4,439	Modifier
Upstream gene variant	4,902	Modifier

Table S3. Variants in genes that expressed a high impact effect

Chromosome no.	Effect	LOC / Gene symbol	Gene Description	Gene type
X	Start lost	LOC115705378	probable transcriptional regulatory protein At2g25830	protein coding
X	Stop gained	LOC115725751	uncharacterized LOC115725751	protein coding
1	Stop gained	LOC115704219	uncharacterized LOC115704219	protein coding

1	Splice donor variant&intron	TRNAY-GUA	transfer RNA tyrosine (anticodon GUA)	tRNA
3	Start lost	LOC115710426	mRNA-uncharacterized LOC115710426	protein coding
3	Stop gained	LOC115710807	uncharacterized protein LOC115710807	protein coding
3	Stop gained	LOC115710807	uncharacterized protein LOC115710807	protein coding
4	Stop gained	LOC115713173	uncharacterized LOC115713173	protein coding
5	Stop gained	LOC115716597	probable calcium-binding protein CML13	protein coding
2	Stop lost	LOC115719224	glycine-rich RNA-binding protein 4, mitochondrial	protein coding
6	Splice acceptor variant&intron	LOC115724884	uncharacterized LOC115724884	ncRNA

Table S4. Distribution of the impact effects across the different number of subcultures

Grouping	High	Low	Moderate	Modifier
Group 6-7	7	1,066	437	16,354
Group 6-8	8	1,076	450	17,500
Group 6-9	8	1,083	457	17,977
Group 6-10	13	1,508	659	23,147
Group 6-11	13	1,508	659	23,149
Group 6-11 & sister line	14	1,516	667	23,602

Table S5. Nucleotide diversity and variants values across the different number of subcultures

Grouping	No. of variants[a]	Avg. no. of variants	No. of sites[b]	Nucleotide Diversity Avg.

Group 6-7	5,385	2.41	2,237	5.19E-05
Group 6-8	6,245	2.58	2,426	4.54E-05
Group 6-9	6,613	2.65	2,501	4.40E-05
Group 6-10	9,072	2.76	3,289	3.45E-05
Group 6-11	9,076	2.76	3,291	3.45E-05
Group 6-11 & sister line	9,405	2.81	3,343	4.08E-05

[a] How many total variants were found
[b] How many locations the variants fell into (20kb range)
